# Health scores for farmed animals: Screening pig health with register data from public and private databases

**DOI:** 10.1371/journal.pone.0228497

**Published:** 2020-02-04

**Authors:** Franziska Nienhaus, Diana Meemken, Clara Schoneberg, Maria Hartmann, Thomas Kornhoff, Thomas May, Sabrina Heß, Lothar Kreienbrock, Anna Wendt

**Affiliations:** 1 Field Station for Epidemiology Bakum, University for Veterinary Medicine Hannover, Zeven, Germany; 2 Institute of Food Safety and Food Hygiene, Working Group Meat Hygiene, Freie Universität Berlin, Berlin, Germany; 3 Department of Biometry, Epidemiology and Information Processing, WHO Collaborating Centre for Research and Training for Health in the Human-Animal-Environment Interface, University for Veterinary Medicine Hannover, Hannover, Germany; 4 QS Qualität und Sicherheit GmbH, Bonn, Germany; University of Illinois, UNITED STATES

## Abstract

There are growing demands to ensure animal health and, from a broader perspective, animal welfare, especially for farmed animals. In addition to the newly developed welfare assessment protocols, which provide a harmonised method to measure animal health during farm visits, the question has been raised whether data from existing data collections can be used for an assessment without a prior farm visit. Here, we explore the possibilities of developing animal health scores for fattening pig herds using a) official meat inspection results, b) data on antibiotic usage and c) data from the QS (QS Qualität und Sicherheit GmbH) *Salmonella* monitoring programme in Germany. The objective is to aggregate and combine these register-like data into animal health scores that allow the comparison and benchmark of participating pig farms according to their health status. As the data combined in the scores have different units of measure and are collected in different abattoirs with possibly varying recording practices, we chose a relative scoring approach using z-transformations of different entrance variables. The final results are aggregated scores in which indicators are combined and weighted based on expert opinion according to their biological significance for animal health. Six scores have been developed to describe different focus areas, such as "Respiratory Health", "External Injuries/ Alterations", "Animal Management", "Antibiotic Usage", "*Salmonella* Status" and "Mortality". These "focus" area scores are finally combined into an "Overall Score". To test the scoring method, existing routine data from 1,747 pig farm units in Germany are used; these farm units are members of the QS Qualität und Sicherheit GmbH (QS) quality system. In addition, the scores are directly validated for 38 farm units. For these farm units, the farmers and their veterinarians provided their perceptions concerning the actual health status and existing health problems. This process allowed a comparison of the scoring results with actual health information using kappa coefficients as a measure of similarity. The score testing of the focus area scores using real information resulted in normalised data. The results of the validation showed satisfactory agreement between the calculated scores for the project farm units and the actual health information provided by the related farmers and veterinarians. In conclusion, the developed scoring method could become a viable benchmark and risk assessment instrument for animal health on a larger scale under the conditions of the German system.

## Introduction

The last decade has seen new impulses and demands to ensure animal health and to improve animal welfare. High animal health and welfare standards, especially for farm animals, are of importance to the public and are increasingly expected by consumers [1–4]. A variety of activities are proof of these tendencies, such as increased research into methods for measurement of animal health and animal welfare [5, 6], the intention to introduce a label for animal welfare or the implementation of new legal requirements for farmers to document the health status of their animals.

To maintain or improve health conditions of food producing animals in the longer term, an essential first step consists of developing methods that allow the assessment of animal health and identify health deficiencies at herd level. The assessment of animal health or welfare is usually based on quantifiable indicators. There are two groups of indicators: resource-based indicators (e.g., floor type, space allocation) and animal-based indicators, reflecting the response of animals as indicators of their welfare and health deficits (e.g., injuries, mortality, morbidity). The present project confines itself to the use of animal-based measures, which have been shown to be preferable for the assessment of pig herds [7].

There are different methods for collecting data on indicators. Frequently, data are collected on the farm, usually by assessing the condition of individual animals, for example, as proposed by the Animal Welfare Protocol^®^ [8]. While this approach can be quite precise, it is rather cost-intensive and time-consuming [9, 10] when used for the assessment of an entire population of farms or to outline a development over time. Thus, this method does not seem viable if a large number of herds are to be monitored on a regular basis. Hence, we chose a different approach by using data already available by routine inspection along the food chain. During the course of the expansion and specialization of companies in agriculture, farm data evaluations have become increasingly important. Regarding animal health and thus the better performance of the animals, meat inspection results especially are an important part of the farm data analysis [11]. On the one hand, comprehensive animal health scores based on register-like data like these can help to identify problems in farm health management. On the other hand, these data could be of use to veterinary offices as a screening tool for detecting farms with potential health problems or to private enterprises as an efficient tool for quality assurance. Therefore, it is necessary to develop discriminative scores, which would allow for a distinction between different groups (in this case farm units, or different points in time, respectively) in terms of their animal health status.

In this paper, the possibility of developing animal health scores based on already existing data from public and private databases registering information that have been collected along the food chain of fattening pigs in Germany are examined. This information is

- meat inspection data (data section A)
- antibiotics consumption data (data section B) and
- *Salmonella* monitoring data (data section C).

As mortality data (data section D) is a very important indicator to describe animal health it is also included in the examination.

To choose a feasible scoring approach, the selected data have been analysed regarding their distribution and associations. For these real data, 1,747 pig farm units throughout Germany are used as an example.

In a next step, the scoring approach was validated. The scores were calculated for 38 project farm units. These scores were compared to the results of a questionnaire for farmers and their veterinarians dealing with the actual health status of the farms.

## Material and methods—Part 1: Score construction

### Data sources

For the development of health scores for fattening pigs, we selected indicators from existing data collections. Data were maintained by QS, which is the scheme owner and vehicle of the "QS Quality Scheme for Food" that covers all stages of the supply chain in Germany. The standards defined by the scheme owner set forth are stringent, verifiable production and marketing criteria. Within the QS scheme, animal owners, abattoirs, wholesalers and food retailers are obliged to participate in monitoring programmes. Pig fatteners are obliged to participate in a *Salmonella*, an antibiotics and a diagnostic data monitoring. All results obtained in the monitoring programmes are recorded and evaluated in the QS database [12].

In this study, selection criteria for the data were their potential relevance to describe pig health, their suitable form, i.e., availability (routine electronic recording preferred), quality (frequency, accuracy, harmonised definition for data collection, etc.) and sufficient variance to generate enough information allowing for a distinction of different pig farms. Sixteen animal-based measures from four different data sections are included in the selection:

Section A includes **data from the official meat inspection at slaughter**. In Germany, post-mortem meat inspection is carried out routinely for each slaughtered pig according to EU legislation [13, 14]. Each recorded lesion can be attributed to a farm unit, an abattoir and the day of slaughter. The results from meat inspection are documented electronically by the veterinary authorities and stored in databases held by the abattoir. Some of the recorded meat inspection codes have been surveyed for many years according to a national administrative regulation in Germany [15] (pneumonia; pleurisy; pericarditis; liver with milk spots), and others were added to the recording practice only recently (for example, intestinal alterations, arthritis, ear and tail lesions) (details of the coding see Table 1). Because of the detailed reporting, for each combination of farm of origin and abattoir, the data for each code can be summarized as a prevalence related to a chosen time span.
Section B includes herd **data on antibiotic consumption**. Since 2014, this documentation has been mandatory according to the 16^th^ amendment of the German Pharmaceuticals Act [16]. In addition, QS is documenting the use of antibiotics in pigs for its members since 2012. Measures on the treatment frequency (TF) per half year, which relates the number of used daily doses (number of days treated × number of active substances applied × number of animals treated) to the farm size (average number of housed animals per age class) are available electronically (for details, see Schaekel et al. [17]).
Section C includes **data from the QS *Salmonella* monitoring system** [18]. For all farmers raising fattening pigs a target number of samples of serum or meat juice, depending on the farm size, is randomly selected, which should be evenly spread over one year. From this, *Salmonella* antibodies were detected via an ELISA-test. The determined measure in the blood or meat juice samples is the optical density (OD). If the OD% value is ≥40, the sample is classified as "positive". With this information, a prevalence per farm unit can be estimated, e.g., per half year.
Section D includes **data on mortality**. Pig farmers are obliged to collect data on mortality by legal regulation in Germany [19]. Until now, these data have not yet been recorded in a standardized manner, and recording systems vary from farm to farm (with paper-based notation, xls-sheets or others being used such as specialized fattening- or feeding software). Therefore, QS does not provide data on mortality for the moment. However, as mortality is one of the most important indicators for animal health, this information must be taken into account for a health assessment. Therefore, a uniformized query of the number of animals which died and were culled and the total number of animals housed per half year for the project farm units was carried out. To calculate the mortality rates, the number of animals which died and were culled was divided by the total number of animals housed per half year.

**Table 1:** QS’s coding scheme for meat inspection at slaughter for pigs.

organ	alteration description
lungs	no alteration
	slightly altered (<10%)
	moderate altered (10–30%)
	highly altered (>30%)
pleura	no alteration
	slightly altered (<10%)
	moderate altered (10–30%)
	highly altered (>30%)
pericardium	no alteration
	altered
liver	free from hepatic milk spots
	milk spots
intestine	no alteration
	inflammation
ear	intact
	not intact (necrosis, inflammation, loss of substance)
tail	no alteration
	necrosis, inflammation
bursa	no alteration
	bursa swelling/bursitis (>5 cm)
dermal damage (handling)	no alteration
	dermal damage because of handling
abscess (partial condemnation)	no alteration
	abscess(es) that leads to condemnation
arthritis (partial condemnation)	no alteration
	joint inflammation/injury that leads to condemnation
dermal alteration (partial condemnation)	no alteration
	large inflammation that leads to condemnation (e.g., scabies)
whole carcass condemnation	no
	unfitted (distinct alterations, e.g., multisystemic wasting)

### General framework of the scoring approach

Data were from individual animals as well as aggregated within farm units. To condense data into a farm-linked animal health score, the following requirements were predefined:

- allow for a holistic view, i.e., provide a simple risk assessment in cases where no details are needed
- be capable of describing different areas of concern in animal health to provide helpful insights for farmers and veterinarians
- provide a comparison of farm units, at best self-explaining benchmarking and
- provide a relative assessment, i.e.,–like the German antibiotics monitoring system–without predefining a fixed threshold or cut-off, but assess farm units in relation to other farm units in Germany.

Therefore, discriminative scores were developed with the purpose of distinguishing farm units according to their health status. To achieve this goal, individual score components (variables) were combined, i.e., an aggregated sum score was calculated using the transformed raw data. As the indicators available differ in their biological significance regarding the description of animal health, they were given different weights based on an expert opinion rating.

To satisfy both requirements (providing a holistic view and a description of specific areas of concern), a two-step approach was followed. In the first step indicators were aggregated in different "focus area scores" reflecting the different aspects of animal health, such as "Respiratory Health" or "External Injuries/ Alterations". As each focus area was described separately, no compensation between these areas occurred. In the next step, the results from the area scores were aggregated into an overall score.

To link the indicators into "focus areas", a factor analysis was applied to an integrated data set including data on meat inspection, data on antibiotic consumption and data from *Salmonella* monitoring. In addition, ten porcine health experts were interviewed regarding the issue of which combination of indicators they considered to be sensible. Scientists as well as specialized pig practitioners working in herd health management and experts from agricultural producer organizations with experience in pig health were involved. The interviews were based on a questionnaire and were carried out by phone or face-to-face.

**Fig 1:**
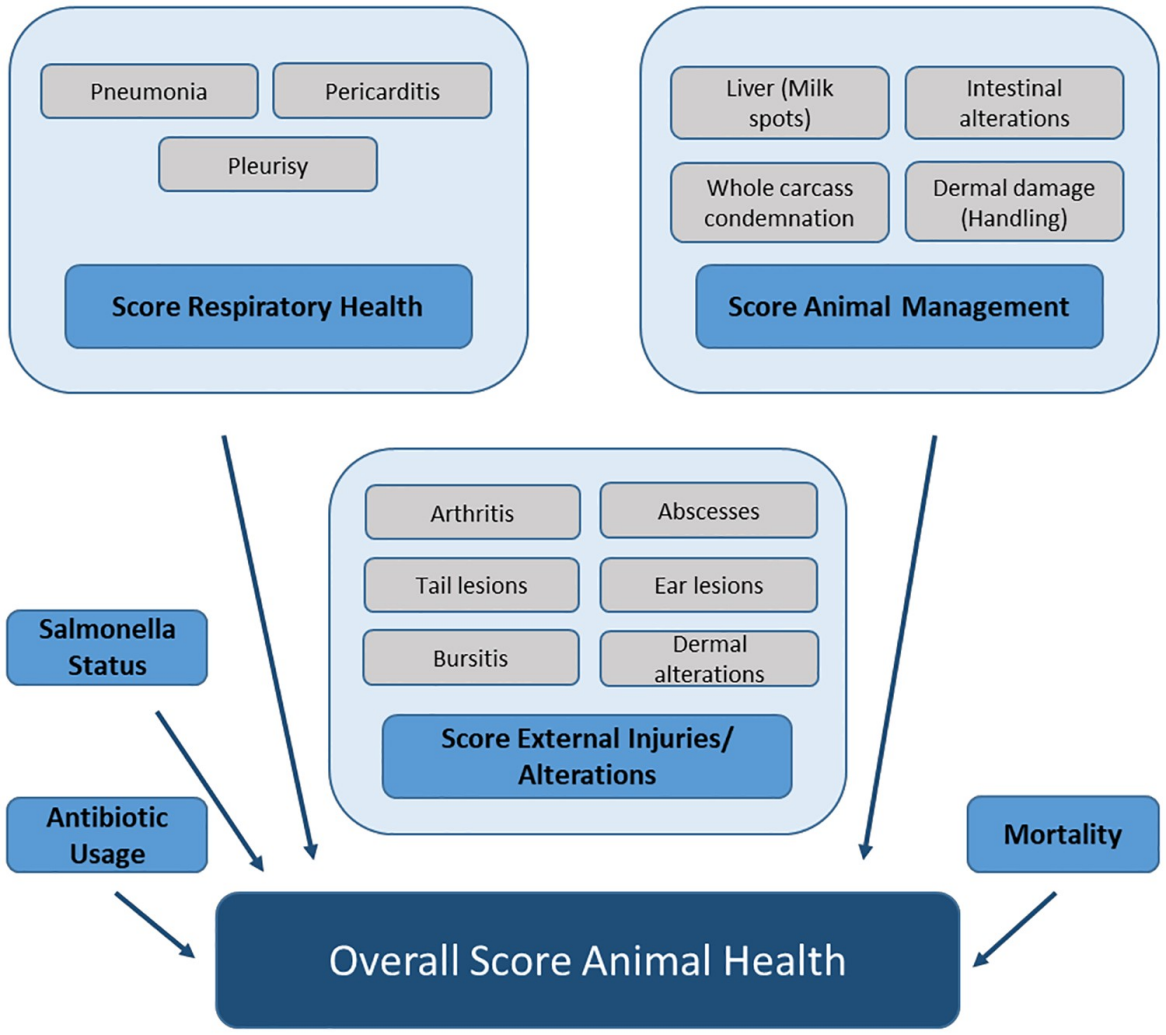
Indicators and scores included to describe health at the farm level.

Six different area scores were defined (Fig 1). Whereas the focus area scores "Respiratory Health", "Animal Management" and "External Injuries/ Alterations" were composed of indicators from the meat inspection, calculation of the scores for "*Salmonella* Status", "Antibiotic Usage", and "Mortality" was based on one single indicator.

### Score definition

Because data per farm unit were from different scales (see data section above), the transformation of all input variables into one harmonised scale was crucial. An additional challenge was the need to compensate for differences in the recording practice of certain indicators at the different abattoirs. Thus, z-standardization was applied to define scores ranging on a symmetric scale around 0 and reach from -∞ to + ∞, i.e., following formula (1) data x from farm i was transformed into

$${\text{z}}_{\text{i}}=\frac{{\text{x}}_{\text{i}}-\bar{\text{x}}}{\text{s}}.$$ (1)

As one condition for the use of a z-standardization in (1) is an approximate normal distribution of the input data, prevalence data p from farm i (like meat inspection data, mortality data and *Salmonella* antibody status) were transformed via logit-transformation, i.e.,

$${\text{x}}_{\text{i}}=\;\text{log}\;\text{it}\left({\text{p}}_{\text{i}}\right)=\text{ln}\left(\frac{{\text{p}}_{\text{i}}}{1-{\text{p}}_{\text{i}}}\right)$$ (2)

and skewed data such as therapy frequency were transformed via an ordinary logarithm to a base of ten.

To calculate the z-values of the treatment frequency and the *Salmonella* antibody status, the means and standard deviations of the log10- or the logit values, respectively, of all counts in our dataset were used. For meat inspection data, the mean and standard deviation of the logits were calculated for each abattoir separately to approach the possible problem of a divergent recording in different abattoirs. To avoid calculation errors within the mathematical transformation for all p < 0.001, the general value 0.001 was set. The same procedure was established for therapy frequencies < 0.01, which were generally set to 0.01.

If a farmer supplied multiple (i.e., two or more) abattoirs per half year, multiple z-values were calculated per indicator (i.e., one z-value assigned to abattoir 1 and one z-value assigned to abattoir 2). As only one value per indicator per farm unit per half year was needed in the end, in such cases, the different z-values per indicator were averaged weighted according to the number of slaughtered animals in the respective abattoir, i.e., for S abattoirs for farm i the calculation is

$${\text{z}}_{\text{i}\;\text{total}}=\sum_{\text{j}=1}^{\text{S}}{\text{w}}_{\text{i}\;\text{j}}\times {\text{z}}_{\text{i}\;\text{j}\;}$$ (3)

with

$${\text{w}}_{\text{i}\;\text{j}}=\frac{\text{no}.\;\text{animals}\;\text{from}\;\text{farm}\;\text{i}\;\text{slaughtered}\;\text{at}\;\text{abbatoir}\;\text{j}}{\sum_{\text{k}=1}^{\text{S}}\text{no}.\;\text{animals}\;\text{from}\;\text{farm}\;\text{i}\;\text{slaughtered}\;\text{at}\;\text{abbatoir}\;\text{k}}.$$

### Aggregation model for the calculation of animal health scores

When a multitude of criteria are combined in one score, the criteria with a larger influence on the described attribute should be assigned greater importance, i.e., a greater weight. As almost no well-evaluated scores for the description of animal health have been documented in the scientific literature, little is known about a viable way of assigning weight to individual indicators. Therefore, experts were asked to note the weights they would give on a five-point Likert scale ranging from 1 (not relevant) to 5 (highly relevant) to the different indicators. We used the median of the experts’ opinions as the weight W_a_ for the following calculations of area scores, if a number of A indicators were given in an area (Table 2). If z_a i total_ denotes the z-score for a given indicator of farm i, summarized in total over all abattoirs, then an area score is calculated using the aggregation model in (4), i.e.,

$$\text{Scor}{\text{e}}_{\text{i}}\;=\sum_{\text{a}=1}^{\text{A}}{\text{W}}_{\text{a}}\times {\text{z}}_{\;\text{a}\;\text{i}\;\text{total}}.$$ (4)

with

$${\text{W}}_{\text{a}}=\frac{\text{Expert}\;\text{Median}\;\text{of}\;\text{Indicator}\;\text{a}}{\sum_{\text{j}=1}^{\text{A}}\text{Expert}\;\text{Median}\;\text{of}\;\text{Indicator}\;\text{j}}.$$

**Table 2:** Selected indicators with their description, mean prevalence or mean treatment frequency, standard deviation, minimum, maximum, and assigned median expert weight for meat inspection indicators.

Data Section	Area	Indicator	Description	Mean (%)	STD	Min (%)	Max (%)	Expert weights
A	Respiratory Health	pneumonia	aberrations according to pneumonia >10%	10.81	10.71	0.00	79.10	5
		pleurisy	aberrations according to pleurisy >10%	6.23	7.79	0.00	74.07	5
		pericarditis	alteration	4.06	4.29	0.00	50.00	4
	External Injuries/Alterations	arthritis	inflammation	1.05	1.83	0.00	30.00	3.5
		abscess	abscess	1.34	2.11	0.00	30.00	3
		ear lesions	necrosis/inflammation	0.26	2.97	0.00	100.00	5
		tail lesions	necrosis/inflammation	0.59	1.53	0.00	20.00	5
		dermal alterations	extensive inflammation	0.27	0.91	0.00	31.67	4
		bursitis	bursitis present	0.51	1.66	0.00	41.10	2.5
	Animal Management	liver (milk spots)	altered with milk spots	14.69	19.00	0.00	95.00	4.5
		dermal damage (handling)	altered through punch marks	0.04	0.76	0.00	30.77	5
		intestinal alterations	inflammation	1.04	2.78	0.00	44.11	1
		whole carcass condemnation	alteration	0.35	1.00	0.00	22.47	1.5
B	Antibiotic Usage	treatment frequency	used daily doses/per housed animals	2.85	5.65	0.00	87.95	-^2^
C	*Salmonella* Status	*Salmonella* status	positive samples in the antibody testing	12.28	14.06	0.00	84.62	-^2^
D	Mortality	mortality	dead and culled animals	-^1^	-^1^	-^1^	-^1^	-^2^

^1^Data according to "Mortality" were only available for the 38 participating project farm units.

^2^"Antibiotic Usage", "*Salmonella* Status" and "Mortality" scores were directly implemented with the z-scores linked to the original data (no expert weights were needed).

Applied to the project herein, the following Table 2 formula (4) turns to specialized area scores in data section A as follows:

- Score_resp_health = 5×Z_total__Pneumonia + 5×Z_total__Pleurisy + 4×Z_total__Pericarditis)/14;
- Score_extern_injuries = (3,5×g_ Z_total__Arthritis + 3× Z_total__Abcess+ 5× Z_total__Ear+5× Z_total__Tail+ 2,5× Z_total__Bursitis + 4× Z_total__Skin)/23
- Score_animal_management = (1×g_ Z_total__Intestine + 5× Z_total__Damage + 4,5× Z_total__Liver + 1,5× Z_total__condemnation)/12 (4 a-c)

For the further calculation of an overall score, the area scores "Respiratory Health", "External Injuries/ Alterations" and "Animal Management" from (4 a-c) were complemented by "Antibiotic Usage", "*Salmonella* Status" and "Mortality" scores directly implemented with the z-scores linked to the original data.

Thus, an overall score was defined by formula (5) as

$$\text{Overall}\;\text{score}=\sum_{\text{Areas}}{\text{W}}_{\text{Area}}\times \text{Scor}{\text{e}}_{\text{Area}}.$$ (5)

To be able to assign a weight to each area score, the ten experts were also asked to rank them according to their importance for animal health. We determined the medians from the experts’ ranking, which were used as a weight attributed to the respective area scores for the calculation of the overall score (Table 3). A ranking of the importance of mortality was not established. As this indicator undoubtedly had very high meaning for the assessment of animal health, it was attributed the maximum weight.

**Table 3:** Expert opinion ranking of area scores for the final overall score.

Area Score	Mean	Median	Minimum	Maximum
Respiratory Health	4.90	5.00	4.00	5.00
External Injuries/Alterations	4.20	4.00	3.00	5.00
Animal Management	4.50	4.50	4.00	5.00
*Salmonella* Status	2.50	2.50	2.00	3.00
Antibiotic Usage	3.50	3.50	3.00	4.00

The following Table 3 formula (5) utilizes

- Score_overall = (5× Score_Respiratory_ + 4×Score_Injuries_+ 4.5×Score_Management_ + 3.5× Z_total__Antibiotics + 2.5× Z_total__Salmonella + 5× Z_total__Mortality) /24.5 (5a)

## Material and methods—Part 2: Score evaluation

As only few experiences have been documented in the domain of score construction related to animal health, the developed scoring method described above has been evaluated twice. First, its feasibility was tested by the application of real live data from the QS routine to show the distribution of original measures, transformed values and final scores. Second, it was validated by comparing the scores with information on the actual health status of selected project farms to verify that the animal health scores developed were valid, i.e., that they reflected the actual health status and enabled farm unit benchmarking.

### Score feasibility testing using routine data

For the calculation of the area scores described above and the overall score, we used existing data on fattening pigs, slaughtered in Germany between 01/07/2016 and 31/12/2016. Data were provided from QS, including data on meat inspection, antibiotic usage and *Salmonella* status. Routine data on mortality were not available. To improve the feasibility of the score calculation, a simple random sample of farm units was selected out of the collective of fattening pig farmers in Germany. Farm-abattoir combinations containing less than ten observations (slaughtered pigs) per half year were excluded in the following analyses. The dataset includes data from 1,747 pig farm units and 58 abattoirs in total. As some farm units supplied more than one abattoir, there are 3,084 combinations of the farm of origin and abattoir in the reported half year.

Data were evaluated with SAS, version 9.4 TS level 1M5 (SAS Institute Inc., Cary, NC, United States) using the mathematical functions included in the software.

### Score validation: Comparing scoring results with on-site health information in a field survey

For a field survey, 38 farm units for a total of 16 farmers, from a pig-dense region in Northwest Germany, were recruited. The farmers were recruited by the producer organization according to a requirement profile predefined by the project partners. The farms should

- be located in one of the two districts involved in the project
- have a close supply relationship with one of the involved abattoirs
- market the majority through the associated producer organization
- have a minimum farm size of 500 fattening places
- be evaluated as an independent company and
- include fattening pigs only.

For these 38 farm units for the second half 2016, all health scores were calculated as described above. Since mortality is not yet recorded by default, it has been collected in a standardized manner for the project farms using a survey sheet of the number of animals kept and the dead and culled animals in the study period. In August 2017, the 16 farmers and ten related veterinary practices were retrospectively interviewed regarding the actual animal health status of each farm unit in the second half of 2016 by using a self-reporting questionnaire developed for this purpose. To validate the newly developed animal health scores, in a next step, the calculated scores and the results of the questionnaire were compared by calculating the concordance index Kappa. This was only done for the overall score animal health and the area scores "Respiratory Health", "External Injuries/Alterations" and "Animal Management" because the other area scores were simply z-transformations from already existing indices.

### Development of a questionnaire on the health status of fattening pig farm units

The questionnaire developed was derived of material and survey sheets from other German doctoral thesis projects [20–24]. All symptom areas that usually occur in fattening pigs in clinical trials, according to Petersen et al. [25], were included. The questionnaires were therefore divided into five different animal health areas: respiratory health, external injuries/alterations, animal health as a whole, animal management, and livestock problems, respectively. Therefore, the various areas of animal health for which a score was developed could be queried in different sets of questions. At the beginning of each questionnaire part, the relevant fattening pig livestock should be classified in the form of a "quarter estimate" of the particular area compared with other fattening pig livestock in the region for the period concerned, i.e.,

- 1^st^ quarter: very good or the best farm units in the respective range
- 2^nd^ quarter: good farm units in the respective range
- 3^rd^ quarter: moderate farm units in the respective range
- 4^th^ quarter: worst farm units in the respective range.

The further questions on the health status of the fattening pig herds in the form of a query of symptoms had to be answered on a Likert-scale with the characteristic values "no", "rare", "occasionally", "frequently" and an additional prevalence estimate in the form of a percentage. The questions of the sets "Animal management" and "Livestock problems" were largely formulated as open questions.

A full version of the original questionnaire in German language can be found online at https://www.tiho-hannover.de/kliniken-institute/institute/bioepi/publikationen/zusatzmaterial-publikationen/.

### Calculation of the concordance index Kappa

The scores based on routine data and the results from the questionnaire were compared by means of concordance measurement. The purpose of calculating the concordance index Kappa in this study is to examine the extent to which the animal health scores can indicate the actual health status of the herd as assessed by the farmers and veterinarians. Here, Kappa refers to the farmers´ and veterinarians’ estimated farm situation (approximated by the quarter estimates from the questionnaire) and the four-fold classification based on the developed animal health scores. The quarter estimates of farmers and veterinarians have been summarized into a common quarter estimate by averaging both answers. The weighting of the kappa coefficient includes the extent of non-conformity of discordant pairs in the calculation.

Data were evaluated with SAS, version 9.4 TS level 1M5 (SAS Institute Inc., Cary, NC, United States) using similarity options within the FREQ procedure.

## Results

### Part 1: Score testing using real routine data

Descriptive statistics for the 16 indicators finally included along with experts’ median weights are provided in Table 2. While the classic meat inspection indicators "pneumonia", "pleurisy", "pericarditis" and "liver with milk spots" showed a mean prevalence of 4% to 14.7%, most of the recently added meat inspection indicators showed a mean prevalence below the 1% level.

**Fig 2:**
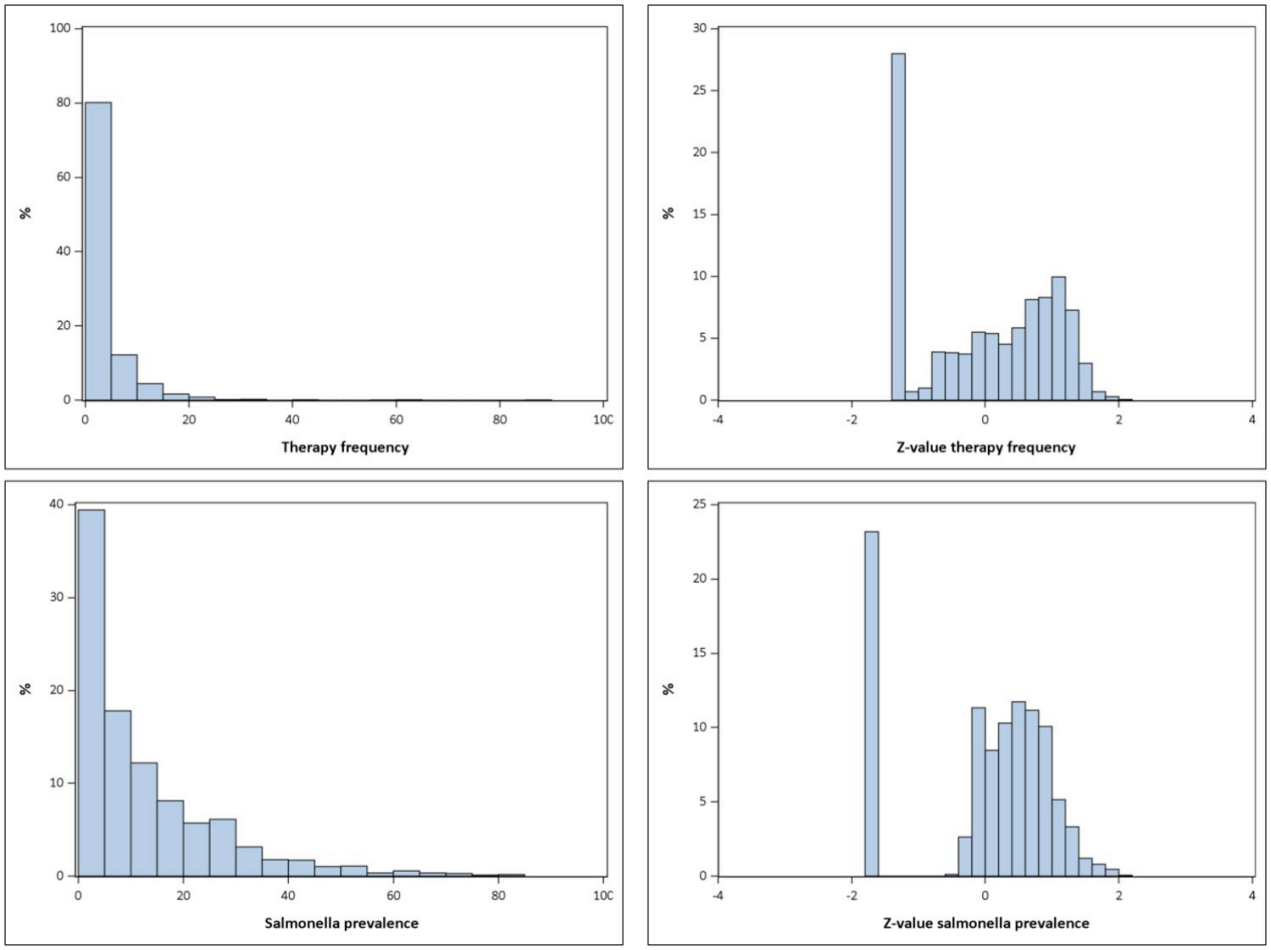
Distribution of therapy frequency and *Salmonella* prevalence and their z-values.

Visual inspection of the distributions of the original values of therapy frequency and the *Salmonella* prevalence per farm unit showed a skewed distribution on the original scale yielded into an approximate normal distribution with zero inflation after the transformation process (Fig 2).

**Fig 3:**
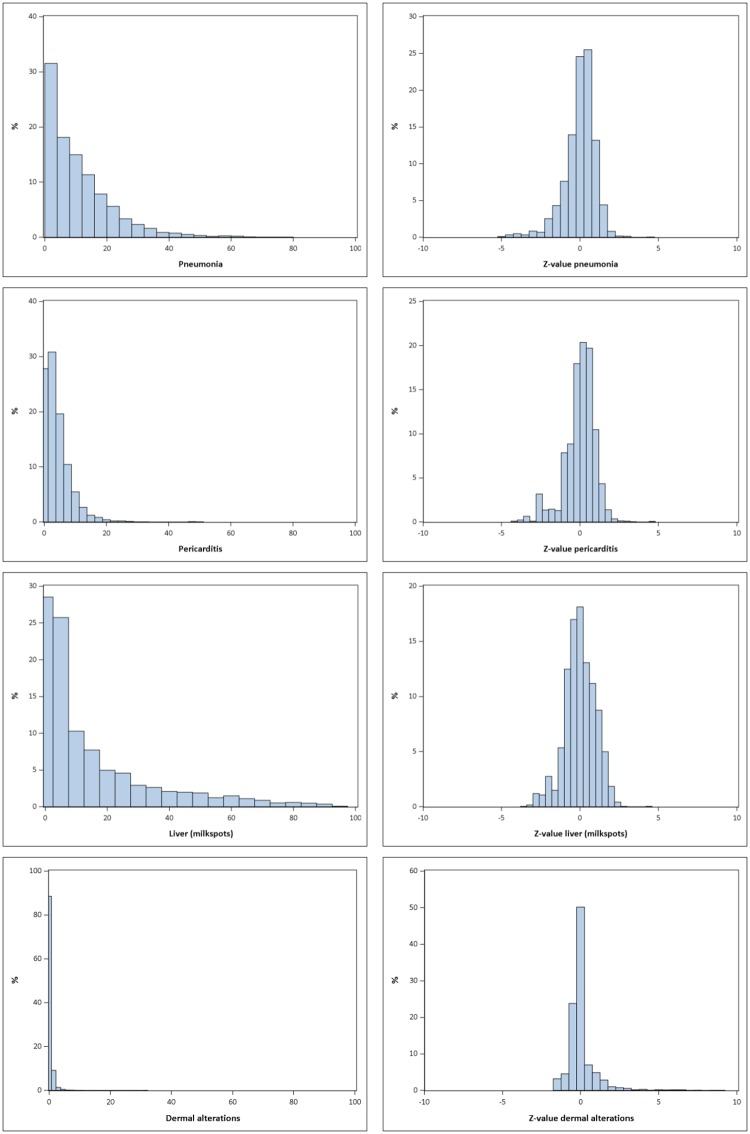
Distribution of the prevalence of meat inspection codes and their z-values for pneumonia, pericarditis, liver and dermal alterations.

This skewed distribution that yielded into an approximate normal distribution with zero inflation was always true for meat inspection data, even in cases of very rare events (Fig 3; a complete description of all distributions analysed can be found online at https://www.tiho-hannover.de/kliniken-institute/institute/bioepi/publikationen/zusatzmaterial-publikationen/).

**Fig 4:**
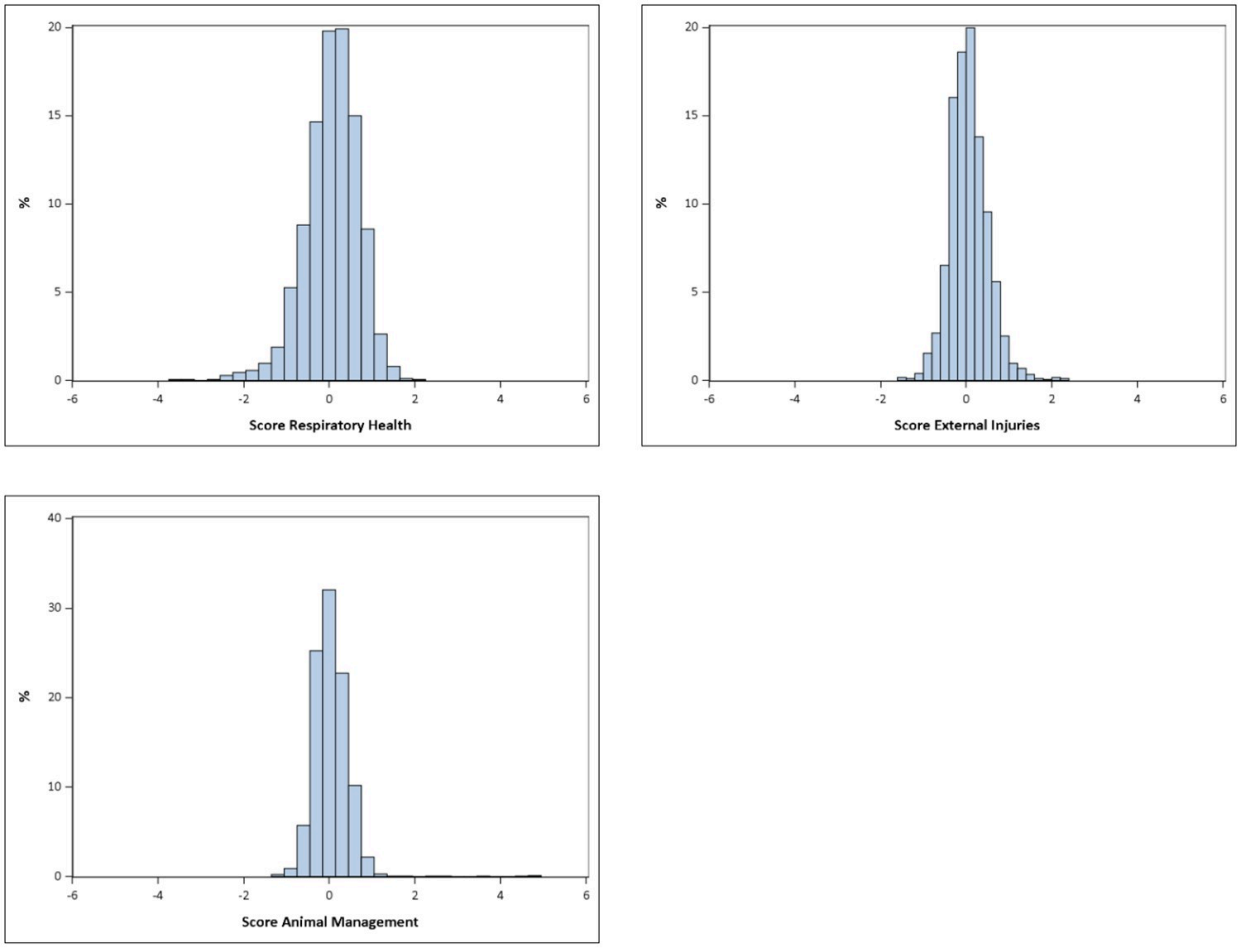
Distribution of score values for Respiratory Health, External Injuries/ Alterations and animal management.

Consequently, calculating the respective area scores and visualizing them for the 1,747 farms selected for this evaluation resulted in distributions that were normally scattered around zero (Fig 4). The scores usually ranged from -3 to +3, with negative scores obtained from farms belonging to the better half of the collective and positive scores given to farms belonging to the inferior half of the population under study.

### Part 2: Score validation: Comparing scoring results with on-site health information

**Fig 5:**
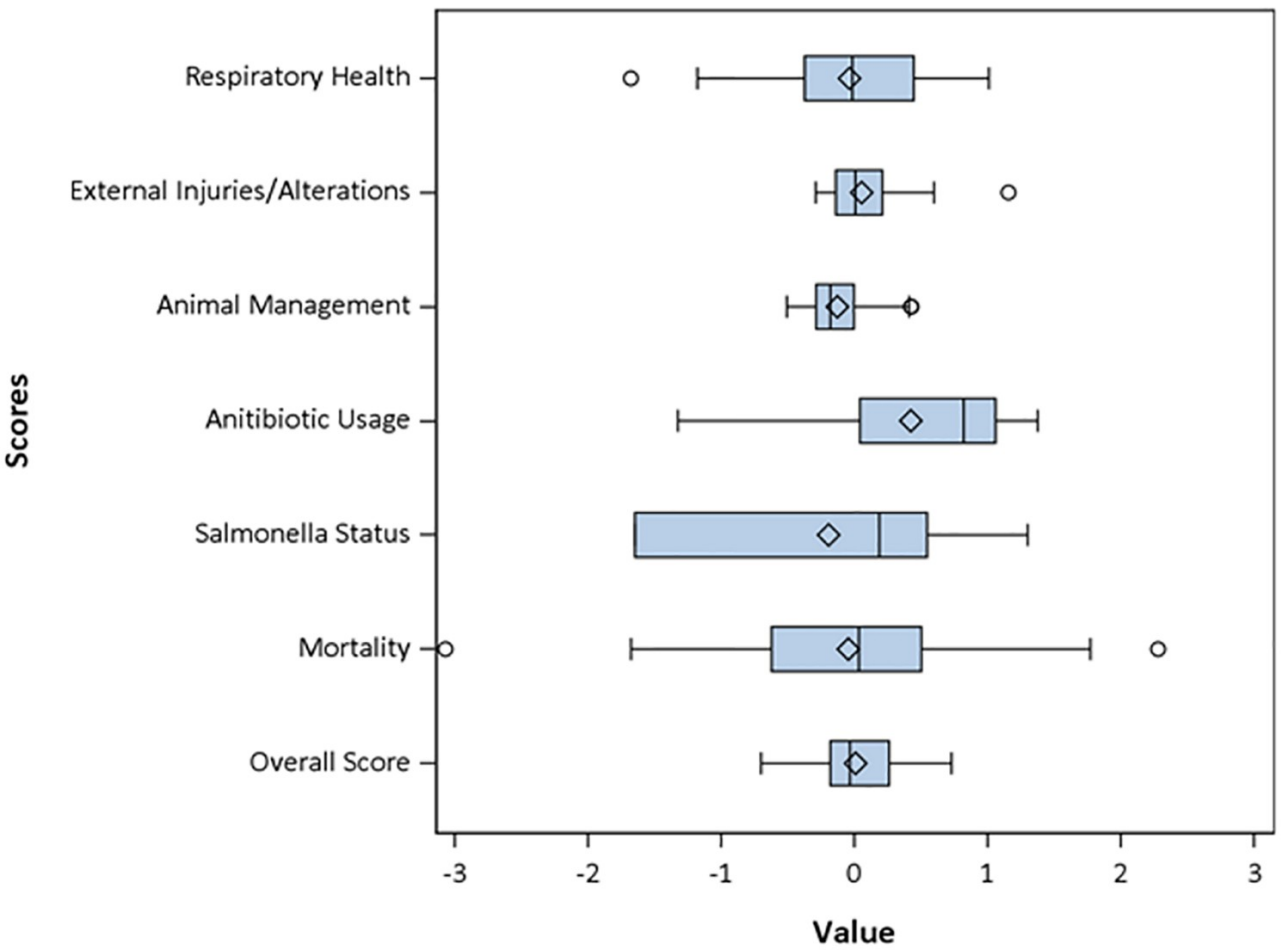
Distribution of the scores of farms included in the field validation exercise.

Convenient sample scores for the 38 project farm units were calculated as described, including a score for mortality for the second half of 2016. For standardization of the frequency of findings per abattoir, which was necessary for the calculation of z-scores, QS provided a further pseudonymized data set. From this dataset for the nine abattoirs supplied by the 16 project partners 1,226 farm unit-abattoir-combinations of 774 farm units with a total of 634,634 slaughtered pigs were available. Overall, there was a very even and symmetric distribution of all scores of the project farm units (Fig 5), which suggested that the farm units selected for the project were a representative cross-section of the entire farm-population as introduced by QS.

If the project farm units selected were classified for each area score in four quarters based on quartile values of the described dataset, for each score farm units could be found in each quarter (Table 4).

**Table 4:** Classification of project farm units based on quartile values of the entire collective; all percentage values are rounded values.

Score Areas	Quarter			
	1	2	3	4
Respiratory Health	12	11	8	7
	31.5%	29%	21.1%	18.4%
External Injuries/ Alterations	8	10	12	8
	21.1%	26.3%	31.6%	21.1%
Animal Management	14	13	6	5
	36.8%	34.2%	15.8%	13.2%
Antibiotic Usage	7	6	13	12
	18.4%	15.8%	34.2%	31.6%
*Salmonella* Status	13	12	9	4
	34.2%	31.6%	23.7%	10.5%
Overall Score Animal Health	11	11	11	5
	29%	29%	29%	13.2%

To provide an overview of the validation of the scoring method, 4×4 similarity tables were calculated for the different scores contrasting the quarter estimates from the questionnaire on self-reported health status with the scoring quarters (Table 5). Taking into account that a general concordance is accepted by scoring within ± 1 quarter for the Overall Score "Animal Health" (grey shaded areas), only 13.2% of the farms appeared to be discordant, suggesting a fairly good scoring quality. For the area scores, only some additional farm units appeared to be discordant by scoring ± 1 quarter. For "Respiratory Health" and "External Injuries/Alterations" discordance was both 21.1%, and for “Animal Management” 18.4%. The good scoring quality was additionally shown by means of the weighted Kappa-coefficients, which indicated that the results of both measurements were in agreement.

**Table 5:** Similarity tables contrasting scores with self-reported animal health (first number frequencies, second number percentages).

**Focus area "Respiratory Health"κ = 0.1282**					
**on site quarter estimates**	**score quarters**				
	**1**	**2**	**3**	**4**	**Total**
**1**	2	0	2	1	5
	5.26	0.00	5.26	2.63	13.16
**2**	6	8	3	1	18
	15.79	21.05	7.89	2.63	47.37
**3**	4	3	3	5	15
	10.53	7.89	7.89	13.16	39.47
**4**	0	0	0	0	0
	0.00	0.00	0.00	0.00	0.00
**Total**	12	11	8	7	38
	31.58	28.95	21.05	18.42	100.00
**Focus area "External Injuries/ Alterations" κ = 0.1455**					
**on site quarter estimates**	**score quarters**				
	**1**	**2**	**3**	**4**	**Total**
**1**	3	1	1	1	6
	7.89	2.63	2.63	2.63	15.79
**2**	4	9	9	5	27
	10.53	23.68	23.68	13.16	71.05
**3**	1	0	1	2	4
	2.63	0.00	2.63	5.26	10.53
**4**	0	0	1	0	1
	0.00	0.00	2.63	0.00	2.63
**Total**	8	10	12	8	38
	21.05	26.32	31.58	21.05	100.00
**Focus area "Animal Management" κ = 0.2159**					
**on site quarter estimates**	**score quarters**				
	**1**	**2**	**3**	**4**	**Total**
**1**	8	2	2	0	12
	21.05	5.26	5.26	0.00	31.58
**2**	6	11	4	5	26
	15.79	28.95	10.53	13.16	68.42
**3**	0	0	0	0	0
	0.00	0.00	0.00	0.00	0.00
**4**	0	0	0	0	0
	0.00	0.00	0.00	0.00	0.00
**Total**	14	13	6	5	38
	36.84	34.21	15.79	13.16	100.00
**Overall Animal Health: κ = 0.1861**					
**on site quarter estimates**	**score quarters**				
	**1**	**2**	**3**	**4**	**Total**
**1**	5	1	0	0	6
	13.16	2.63	0.00	0.00	15.79
**2**	5	7	9	4	25
	13.16	18.42	23.68	10.53	65.79
**3**	1	3	2	1	7
	2.63	7.89	5.26	2.63	18.42
**4**	0	0	0	0	0
	0.00	0.00	0.00	0.00	0.00
**Total**	11	11	11	5	38
	28.95	28.95	28.95	13.16	100.00

In addition, the accordance with a dichotomous division into very good farms (1st quarter) and farms with an impairment of animal health (2^nd^ to 4^th^ quarter) was examined (Table 6). Since it was not possible to weight the strength of the non-match in a dichotomous classification, in this case, the match was estimated using the unweighted kappa coefficients. On such occasions, the kappa coefficients indicated a higher agreement between the two methods.

**Table 6:** Kappa coefficients for a dichotomous division (% = concordance; p = p-value for the McNemar test of similarity).

Focus area score	Kappa	%	p
Respiratory Health	0.0608	65.8	0.0522
External Injuries/ Alterations	0.3028	79.0	0.4795
Animal Management	0.4172	73.7	0.5271
Overall Score Animal Health	0.4825	81.58	0.0588

The score "Respiratory Health" achieved a low Kappa coefficient (0.0608) with the dichotomous classification. However, the concordance with 65.79% concordant pairs nevertheless showed a consensus between the two methods. The Kappa coefficient did not reflect this agreement because of an unequal distribution of the results. As shown in Table 7, there are 23 matches for the 2^nd^ to 4^th^ quarter categories but only two matches for the 1^st^ quarter category. In addition, there was an uneven marginal distribution, which led to the low Kappa coefficient.

**Table 7:** Contingency table for the dichotomous division of the score "Respiratory Health" (first number frequencies, second number percentages).

Focus area score "Respiratory Health"			
	score classification		
quarter estimates	1	2–4	Total
**1**	2	3	5
	5.26	7.89	13.16
**2–4**	10	23	33
	26.32	60.53	86.84
**Total**	12	26	38
	31.58	68.42	100.00

## Discussion

An important goal of the European Food Safety Authority (EFSA) is to make animal welfare measurable as a precondition for animal welfare improvements. However, "The aim of EFSA to assess the welfare of the animals by using a quantitative risk assessment methodology has not been achieved yet" [26]. Animal welfare and animal health scoring is therefore a matter of general scientific discussion [6]. Some of the scores or indices proposed are composed of animal based variables that are levied on the farm, for example, the "Real Welfare" scheme [27]. However, continuous on-farm assessment is time-consuming and expensive and therefore not feasible on a nationwide scale.

Therefore, our aim was to develop animal health/ animal welfare scores from data that are already available and are retrievable electronically for later use as risk assessment and benchmarking tool. In Denmark and Sweden for example, there are similar approaches to measure animal welfare considering existing routine data [28–31]. Although various EU regulations have established framework conditions for data collection along the pork food chain, the data situation differs from country to country because of different national legislation and different private organization labels that collect different health data. Therefore, detailed animal health scores can only be established at the national level.

### Part 1: Indicators included and score definition

In this paper, we have introduced a secondary data analysis method to assess animal health. Each of the secondary data collections used for this purpose has its challenges. Therefore, a fundamental understanding of the available data is necessary for a viable aggregation and transformation of the raw data for its final use. In particular, these challenges concern the meat inspection data. Although EFSA highlights meat inspection data as an important but "under-utilized" surveillance tool for animal health and welfare, it also mentions the specific challenges associated with the sensitivity of these data [32]. For Germany, the studies from Steinmann et al. [33] and Hoischen-Taubner et al. [34] confirm these challenges. One recurrent topic are the differences in the recording practices between different abattoirs. Therefore, the standardization of meat inspection is underway in almost all slaughterhouses in Germany. As part of this process, QS set up a standard catalogue for inspection [35] (Table 1), which will increase the precision of the data in the long run. The formal codes used by QS for the post-mortem meat inspection were supplemented by new findings on 01.07.2016 [35]. For all abattoirs that participate in the QS system, this obligation has only applied since 01.01.2018 [36]. Therefore, as expected, the quality of the data will steadily improve in the near future.

Thus, overall, the data used have general variability. Because these data are on a quantitative scale, the risk of generating health/ welfare scores with false-positive or false-negative results increases. In terms of an exposure-score relation between farm characteristics and other drivers of the developed scores, an information bias may arise. However, for this relation, a non-differential bias has to be stated, i.e., all possible relations are diluted to the Null, which, conversely, indicates a lower bound of causality if the scoring identifies a relation as such [37].

Beside the type of data included, a number of decisions had to be made prior to the actual score construction, e.g., regarding the scoring approach and the scaling level of the final scores. As a reaction to time-dependent fluctuation, and due to further characteristics of the available data (such as the frequency of data collection or the amount of available data), the time-span chosen to calculate the health scores was half a year, which is directly linked to the legal requirements of the German pharmaceutical act [16].

In our project, we used the interval level health scores, which allowed to obtain quantitative information on animal health that permitted a differentiated comparison of farms [2]. As the underlying input variables (prevalence data and therapy frequency) were continuous, a decision for ordinally scaled scores would imply a loss of information.

Due to the characteristics of the available routine data, an approach was chosen in which the score value described the health status in a relative manner. This approach was found in two elements of the calculation of the score: first in the calculation of the z-values per indicator per abattoir; second, in the interpretation of the final area and overall scores.

Transforming the prevalence data from meat inspection into z-values allows compensation for the effect that data from different abattoirs show different prevalence levels. This latter phenomenon has been frequently described [38–41] and was confirmed in our pre-analyses. It is not possible to identify the source of "abattoir-associated" variation with the kind of data that are available for our analyses. As a qualified guess, variation might occur due to different recording practices and technical surroundings at the abattoir or to a difference in the real prevalence of disease between the subset of farms that send their pigs to a specific abattoir. If the cause is a difference in the recording practices of different abattoirs, different approaches of harmonisation and training might be applied to deal with the situation. It should be noted that it usually takes years to establish a stable, functioning and consistent system, which generates reliable and comparable data.

Regarding the relative interpretation of the final scores as opposed to an absolute interpretation, this approach is helpful if there is no absolute threshold available for a classification into "good" or "bad". For indicators such as dermal alterations as an example, which in Germany have been added to meat inspection only recently, knowledge regarding the actual prevalence in fattening pigs is therefore scarce; thus, the indicators cannot be assessed using an absolute threshold defined in advance. This approach is similar to the legal German approach in antibiotics monitoring [16].

It must be noted that, during this procedure, a score value calculated for a certain farm unit at a certain moment in time cannot be compared with the later score value directly due to the temporal variation within the population, which will shift the relative position of a single farm even, if no changes in the absolute health outcome take place over time. Thus, this approach permits comparisons of the health status calculated over a given period of different farms, but it might be incorrect when used to describe the development of one farm’s health status over time. However, the relative scoring can indicate whether a farm has a better or worse ranking than other farms in comparison to a previous period. This contributes to a continuous improvement in animal health, as the definition of good animal health in this scoring approach always depends on the entire collective, which is expected to improve as a result of benchmarking. To accompany this relative scoring, the absolute temporal development of specific animal health indicators within one farm is therefore crucial. In addition, this helps to avoid "overaggregating", which may occur, if some area scores yield into a good rating and some into a bad rating of a farm, which at the end yield into a normal rating of the overall score.

Regarding the weighting of indicators, expert opinions were used in this publication. Weightings inherently imply judgements of what is more important and consequential. These judgements might be subject to change, and therefore, weights should be revised over time or by an extended group of experts.

### Part 2: Score evaluation

The data presented in this paper evaluated the score definition in two directions. First, the scoring method was evaluated in terms of its feasibility by using routine data from 1,747 pig farm units and 58 slaughterhouses. The results showed that the scores were distributed normally scattered around zero, which indicated that the scores covered a huge range of possible outcomes and would be able to benchmark farms within this approach.

Second, the method was tested directly in an evaluation study, including 38 farm units, for which the scores were considered. In addition, the farmer’s and veterinarian’s view were both taken into account. It was expected that especially the very dedicated farmers would be willing to participate in a project that involves the networking of data for the purpose of benchmarking. Nevertheless, it was assumed that, similar to the study by Dickhaus et al. [42], animal health data are already expanding from this narrow selection of dedicated farmers from one region belonging to the same producer group. This expansion was very important for confirming whether the scores were appropriate for all categories of animal health. The results of the analysis of the animal health data showed that despite the narrow selection of dedicated farmers in one region, there was a wide spread of the data and a very even and symmetric distribution of the scores over the participating farm units in all score ranges. Therefore, although the study population was constructed as a convenience sample, it must be stated as representative for the entire target population of German pig fattening farms. The classification of the 38 project farm units on the basis of the quartile values of the QS dataset of 774 farm units confirmed this once more, since farm units were located in every quarter.

To our knowledge, studies that validate animal health scores based on information provided by farmers and farm veterinarians about the state of health of a herd are rare. To promote a continuous improvement of animal health and provide a benchmark, the developed animal health scores were dependent on a larger collective. Thus, it was necessary to arrange farm units concerning their health status in quarters. Based on clinical investigations of the project farm units, it would only have been possible to compare them with each other, but we would not have been able not compare them to the rest of the collective on which the score calculation was made. In addition, clinical examinations would always be snapshots of the health status. Therefore, the questionnaire attempted to classify the farm units based on retrospective assessment of the health status over a six-month period in form of quarter estimates. The region in which the project was started is a very pig-dense region, so the project farmers are in close contact and exchange with other farmers with fattening pigs. In addition, many farmers in Germany are in consultancy circles and producer organizations in which a lively exchange is operated. As a result, it is assumed that farmers can rank their farm units in comparison to the rest of the region. To obtain a comprehensive assessment of animal health, the responsible veterinarians were also interviewed. The veterinarians have deep insight into the state of health of all farms they are supervising and are in contact with other veterinarians. Therefore, it was assumed that they were also able to rank the farms in comparison to the rest of the region. Thus, although no direct clinical assessment was available, the estimates of farmers and veterinarians were based on a high degree of reliability.

The comparison of similarity has shown concordance between the quarter estimates from the questionnaire and the quarter division based on the developed animal health scores. The concordance was even better with a dichotomized classification into very good farms and farms with an impairment of animal health. The disparate marginal distributions between quarter estimations related to respiratory health and the quarter division based on the score "Respiratory Health" were due to the dichotomous division into very good farms (1^st^ quarter) and farms with an impairment of animal health (2^nd^ to 4^th^ quarter). Pathogens of respiratory diseases in pig holdings are wide-spread. In pig-dense regions, respiratory diseases are therefore a frequent phenomenon. In addition, respiratory diseases are also usually a cost-intensive problem and therefore gain greater importance, potentially making it difficult for farmers to make a quarter estimate and resulting in an overestimation of the problem. Overall, due to the quarter estimate based on the questionnaire, only five farms were classified into the first quarter. With a small size like this, a low Kappa value does not necessarily indicate low agreement [43]. In this case, there was at least agreement with 65.79% concordant pairs.

In other studies, scores on farm evaluations were mostly obtained using the Welfare Quality^®^ Protocol. Knage-Rasmussen et al. [29] have developed a welfare score based on previously available data for sows. They validated it on the basis of an on-farm welfare index inspired by the Welfare Quality^®^ Protocol and concluded that there was no similarity between the two methods. However, they also mentioned that the on-farm index reflects the situation of one day and the database score of a whole year, so "the two types of data do not cover the same animals in the same environment" [29]. Otten et al. [30] have chosen a similar approach, comparing an index consisting of previously available register-data weighted based on expert opinion with an on-farm index and an index consisting of resource-based measures. In addition, different periods of time were examined for the register-data index. However, no significant similarities were found in their study. To overcome these discrepancies between a long-term score and a one-day herd visit, we have introduced a much more comprehensive method to evaluate animal health scores by both farmers and veterinarians, which shows fairly good similarity. Overall, the method proposed herein seems to be suitable as a benchmarking approach within the pork production chain.

## Conclusion

With these cautionary notes, we suggest that an approach for the calculation of health scores, such as the one described, could be used to assemble data from different abattoirs, data on antibiotics usage, *Salmonella* status and data on mortality, to provide health scores at the farm level as a comprehensive overview under the conditions of the German pork production system. Based on the actuality of the above mentioned changes in the feedback system of animal health and welfare data, a longitudinal re-validation of the scores and especially its weighting is necessary.

In summary, this scoring approach is not to replace a clinical examination, but to provide a tool for risk-oriented control or advice, thus contributing to a continuous improvement of animal health and animal welfare. Because a total score can disguise single areas of animal health, different area scores could be used to draw attention to certain problem areas of animal health. Since the scores are composed of individual data, it is also important to provide these data on the individual animal health indicators for the farmers so that they can carry out causality research together with their veterinarian.

## Supporting information

S1 Supplement Original questionnaire for on-farm evaluation in German language (see https://www.tiho-hannover.de/kliniken-institute/institute/bioepi/publikationen/zusatzmaterial-publikationen/). (PDF)

S2 Supplement Distribution of the prevalence of meat inspection codes and their z-values in a random sample of 1,747 German pig farms in the second half of 2016 (see https://www.tiho-hannover.de/kliniken-institute/institute/bioepi/publikationen/zusatzmaterial-publikationen/). (PDF)

## Abbreviations

AFFL: Working Group on Meat Hygiene and Subject Specific Questions of Foodborne Animal Products
EFSA: European Food Safety Authority
OD: optical density
QS: QS Qualität und Sicherheit GmbH
TF: treatment frequency
