# Supplementary material for: Health scores for farmed animals: Screening pig health with register data from public and private databases
Source: PLoS One. 2020 Feb 4;15(2):e0228497. doi: 10.1371/journal.pone.0228497 (PMC6999879; doi:10.1371/journal.pone.0228497)
Supplement: S1 Supplement — (PDF) [file pone.0228497.s001.pdf]

Supplement 1: Original questionnaire for on-farm evaluation in German language

A Identifikation

A1 VVVO-Nr. / Betriebsname:

____

A2 Datum der Befragung:

____

A3 Name Interviewer:

____

A4 Name Befragter:

____

Der nachfolgende Fragebogen ist unterteilt in 5 verschiedene Fragenblöcke:

B. Respiratorische Gesundheit
C. Äußere Verletzungen/ Veränderungen
D. Tiergesundheit gesamt
E. Tiermanagement
F. Bestandsprobleme

Jeweils zu Anfang der Fragenblöcke B – E soll der oben identifizierte Mastschweinebestand zu verschiedenen Tiergesundheitsaspekten im Vergleich zu anderen Mastschweinebeständen der Region eingeordnet werden.

Dabei bedeutet:
1. Viertel: (die 1. 25%) sehr gute bzw. die besten Betriebe in dem jeweiligen Bereich
2. Viertel: (die 2. 25%) gute Betriebe in dem jeweiligen Bereich
3. Viertel: (die 3. 25%) mäßige Betriebe in dem jeweiligen Bereich
4. Viertel: (die 4. 25%) schlechteste Betriebe in dem jeweiligen Bereich

Bei einigen Fragen wird zusätzlich zu den Antwortmöglichkeiten um eine Prävalenzschätzung (Schätzung der Krankheitshäufigkeit) gebeten. Hier ist eine Prozentangabe erforderlich und in die farblich grün hervorgehobenen Felder einzutragen.

B Respiratorische Gesundheit (Atemwegsgesundheit)

Die Atemwegsgesundheit Ihrer Mastschweine das 2. Halbjahr 2016 rückblickend betrachtend:

B1 Wo würden Sie Ihren Mastschweinebestand im Vergleich zu den anderen Mastbetrieben der Landkreise Vechta und Cloppenburg einordnen?

☐ 1. Viertel ☐ 2. Viertel ☐ 3. Viertel ☐ 4. Viertel

☐ Ich möchte dazu keine Einschätzung abgeben.

☐ Ich kann dazu keine Einschätzung abgeben, weil ich es nicht weiß.

____

B2 Zeigten Ihre Mastschweine Symptome einer Atemwegserkrankung (Niesen, Nasenausfluss, Husten, Atemnot)?

☐ nein **Prävalenzschätzung:** ☐

☐ selten

☐ gelegentlich

☐ häufig

☐ Ich möchte dazu keine Einschätzung abgeben.

☐ Ich kann dazu keine Einschätzung abgeben, weil ich es nicht weiß.

____

Supplement 1: Original questionnaire for on-farm evaluation in German language

Stand: 25.07.2017	PPP-InfoS Fragebogen Landwirt	Fragebogen ID: **L -**

C Äußere Verletzungen/ Veränderungen

Das Auftreten von äußeren Verletzungen/Veränderungen (wie z.B. Schwanzverletzungen, Hauterkrankungen oder Abszesse) Ihrer Mastschweine das 2. Halbjahr 2016 rückblickend betrachtend:

C1 Wo würden Sie Ihren Mastschweinebestand im Vergleich zu den anderen Mastbetrieben der Landkreise Vechta und Cloppenburg einordnen?

☐ 1. Viertel ☐ 2. Viertel ☐ 3. Viertel ☐ 4. Viertel

☐ Ich möchte dazu keine Einschätzung abgeben.

☐ Ich kann dazu keine Einschätzung abgeben, weil ich es nicht weiß.

C2 Zeigten Ihre Mastschweine folgende Veränderungen:

Nabelbrüche

☐ nein ☐ selten ☐ gelegentlich ☐ häufig

Prävalenzschätzung: ☐

Ohrrandnekrosen

☐ nein ☐ selten ☐ gelegentlich ☐ häufig

Prävalenzschätzung: ☐

Schwanznekrosen

☐ nein ☐ selten ☐ gelegentlich ☐ häufig

Prävalenzschätzung: ☐

Stand: 25.07.2017	PPP-InfoS Fragebogen Landwirt	Fragebogen ID: **L -**

Flankenbeißen

☐ nein ☐ selten ☐ gelegentlich ☐ häufig

Prävalenzschätzung: ☐

Gelenksentzündungen

☐ nein ☐ selten ☐ gelegentlich ☐ häufig

Prävalenzschätzung: ☐

Liegebeulen (mit Durchmesser > 5cm)

☐ nein ☐ selten ☐ gelegentlich ☐ häufig

Prävalenzschätzung: ☐

Abszesse

☐ nein ☐ selten ☐ gelegentlich ☐ häufig

Prävalenzschätzung: ☐

Hauterkrankungen

☐ nein ☐ selten ☐ gelegentlich ☐ häufig

Prävalenzschätzung: ☐

Generell:

☐ Ich möchte dazu keine Einschätzung abgeben.

☐ Ich kann dazu keine Einschätzung abgeben, weil ich es nicht weiß.

Supplement 1: Original questionnaire for on-farm evaluation in German language

Stand: 25.07.2017	PPP-InfoS Fragebogen Landwirt	Fragebogen ID: L -

D Tiergesundheit gesamt

Die Gesundheit Ihrer Mastschweine <u>als Gesamtzustand</u> für das 2. Halbjahr 2016 rückblickend betrachtend:

D1 Wo würden Sie Ihren Mastschweinebestand im Vergleich zu den anderen Mastbetrieben der Landkreise Vechta und Cloppenburg einordnen?

☐ 1. Viertel ☐ 2. Viertel ☐ 3. Viertel ☐ 4. Viertel

☐ Ich möchte dazu keine Einschätzung abgeben.

☐ Ich kann dazu keine Einschätzung abgeben, weil ich es nicht weiß.

D2 Hatten Sie in Ihrem Mastschweinebestand Probleme mit Kümmerern?
(Definition: unterdurchschnittliches Wachstum + schlechter Gesundheitszustand)

☐ nein **Prävalenzschätzung:** ☐

☐ selten

☐ gelegentlich

☐ häufig

☐ Ich möchte dazu keine Einschätzung abgeben.

☐ Ich kann dazu keine Einschätzung abgeben, weil ich es nicht weiß.

D3 Hatten sie Probleme mit ungleichmäßigem Wachstum (Auseinanderwachsen) der Tiere

☐ nein **Prävalenzschätzung:** ☐

☐ selten

☐ gelegentlich

☐ häufig

☐ Ich möchte dazu keine Einschätzung abgeben.

☐ Ich kann dazu keine Einschätzung abgeben, weil ich es nicht weiß.

D4 Zeigten Ihre Mastschweine Symptome einer Durchfallerkrankung?

☐ nein **Prävalenzschätzung:** ☐

☐ selten

☐ gelegentlich

☐ häufig

☐ Ich möchte dazu keine Einschätzung abgeben.

☐ Ich kann dazu keine Einschätzung abgeben, weil ich es nicht weiß.

D5 Zeigten Ihre Mastschweine Symptome einer Gelenkserkrankung (Lahmheit, dicke Gelenke)?

☐ nein **Prävalenzschätzung:** ☐

☐ selten

☐ gelegentlich

☐ häufig

☐ Ich möchte dazu keine Einschätzung abgeben.

☐ Ich kann dazu keine Einschätzung abgeben, weil ich es nicht weiß.

D6 Zeigten Ihre Mastschweine Symptome einer Erkrankung des Nervensystems
(z.B. Kreisbewegungen, Rudern in Seitenlage, Krämpfe)?

☐ nein **Prävalenzschätzung:** ☐

☐ selten

☐ gelegentlich

☐ häufig

☐ Ich möchte dazu keine Einschätzung abgeben.

☐ Ich kann dazu keine Einschätzung abgeben, weil ich es nicht weiß.

Supplement 1: Original questionnaire for on-farm evaluation in German language

D7 Welche Krankheitserreger wurden im 2. Halbjahr 2016 bei Ihren Mastschweinen nachgewiesen? (Mehrfachnennung möglich)

Erreger von Atemwegserkrankungen:

☐ APP ☐ HPS ☐ Mykoplasmen (M. hyo.)

☐ Influenzaviren ☐ PRRSV ☐ PCV2

☐ Streptokokken ☐ Pasteurellen ☐ Bordetellen

☐ keine ☐ ich weiß nicht ☐ Andere: _______________

Erreger von Darminfektionen

☐ Lawsonien ☐ Brachyspiren ☐ Salmonellen

☐ E. coli ☐ Rotaviren ☐ Coronaviren

☐ keine ☐ ich weiß nicht ☐ Andere: _______________

Erreger von Gelenkerkrankungen

☐ HPS ☐ Streptokokken ☐ Staphylokokken

☐ E. coli ☐ M. hyorhinis ☐ M. hyosynoviae

☐ keine ☐ ich weiß nicht ☐ Andere: _______________

Erreger anderer Erkrankungskomplexe

☐ keine ☐ ich weiß nicht ☐ Welche: _______________

Generell:

☐ Ich möchte dazu keine Einschätzung abgeben.

☐ Ich kann dazu keine Einschätzung abgeben, weil ich es nicht weiß.

Stand: 25.07.2017	PPP-InfoS Fragebogen Landwirt	Fragebogen ID: L -

E Tiermanagement

Ihr Tiermanagement (z.B. Handling, Biosicherheit, Entwurmungskonzept) das 2. Halbjahr 2016 rückblickend betrachtend:

E1 Wo würden Sie sich im Vergleich zu den anderen Mastbetrieben der Landkreise Vechta und Cloppenburg einordnen?

☐ 1. Viertel ☐ 2. Viertel ☐ 3. Viertel ☐ 4. Viertel

☐ Ich möchte dazu keine Einschätzung abgeben.

☐ Ich kann dazu keine Einschätzung abgeben, weil ich es nicht weiß.

E2 Fahren Sie in Ihrem Bestand ein Rein-Raus-System?

☐ Ja, pro Stall

☐ Ja, pro Abteil

☐ Nein

☐ Ich möchte dazu keine Einschätzung abgeben.

☐ Ich kann dazu keine Einschätzung abgeben, weil ich es nicht weiß.

Supplement 1: Original questionnaire for on-farm evaluation in German language

Stand: 25.07.2017	PPP-InfoS Fragebogen Landwirt	Fragebogen ID: L -

---

Die nachfolgenden Fragen werden offen formuliert.
Die Antwortmöglichkeiten werden nicht mit verlesen.
Anhand der Antwort des Befragten entscheidet der Interviewer, ob eine der vorformulierten Antwortmöglichkeiten zutreffend ist oder ob ein Freitextfeld genutzt werden sollte.

E3 Wie oft reinigen Sie Ihren Stall?

- ☐ Nach jedem Durchgang
- ☐ Ab und zu
- ☐ Andere Antwort:_______________
- ☐ Nein
- ☐ Ich möchte dazu keine Einschätzung abgeben.
- ☐ Ich kann dazu keine Einschätzung abgeben, weil ich es nicht weiß.

E4 Wie oft desinfizieren Sie Ihren Stall?

- ☐ Nach jedem Durchgang
- ☐ Ab und zu
- ☐ Andere Antwort:_______________
- ☐ Nein
- ☐ Ich möchte dazu keine Einschätzung abgeben.
- ☐ Ich kann dazu keine Einschätzung abgeben, weil ich es nicht weiß.

Stand: 25.07.2017	PPP-InfoS Fragebogen Landwirt	Fragebogen ID: L -

E5 Haben sie eine Absonderungsmöglichkeit für kranke Tiere?

- ☐ Ja, einen Krankenstall
- ☐ Ja, ein Krankenabteil
- ☐ Ja, eine Krankenbucht
- ☐ Andere Antwort: ____________
- ☐ Nein
- ☐ Ich möchte dazu keine Einschätzung abgeben.
- ☐ Ich kann dazu keine Einschätzung abgeben, weil ich es nicht weiß.

E6 Wie oft beobachten Sie Ihre Tiere?

- ☐ <1x/Tag ☐ 1x/Tag ☐ 2x/Tag ☐ >2x/Tag
- ☐ Andere Antwort: ____
- ☐ Ich möchte dazu keine Einschätzung abgeben.
- ☐ Ich kann dazu keine Einschätzung abgeben, weil ich es nicht mehr weiß.

E7 Treiben Sie Ihre Tiere dabei auf? ☐ Ja ☐ Nein

E8 Wie ist Ihr Entwurmungsmanagement bzw. wurden Ihre Tiere vor der Eistallung in die Mast entwurmt?

- ☐ Der Ferkelaufzüchter entwurmt während der Aufzucht und dies halte ich für ausreichend.
- ☐ Ich entwurme standardmäßig während der Mast.
- ☐ Wenn beim letzten Durchgang viele Lebern verworfen wurden, entwurme ich beim darauffolgenden Durchgang.
- ☐ Ich habe kein Entwurmungsmanagement
- ☐ Andere Antwort: ______________________________
- ☐ Ich möchte dazu keine Einschätzung abgeben.
- ☐ Ich kann dazu keine Einschätzung abgeben, weil ich es nicht weiß.

Supplement 1: Original questionnaire for on-farm evaluation in German language

---

E9 Wie stehen Sie zum Thema Diagnostik?

- ☐ Diagnostik halte ich für wichtig und frage den Tierarzt danach.
- ☐ Diagnostik möchte ich eher vermeiden, lasse mich aber von meinem Tierarzt beraten.
- ☐ Diagnostik kommt für mich nur im Notfall in Frage.
- ☐ Diagnostik ist mir zu teuer und hat zu wenig Aussagekraft
- ☐ Andere Antwort: ___________________________
- ☐ Ich möchte dazu keine Einschätzung abgeben.
- ☐ Ich kann dazu keine Einschätzung abgeben, weil ich es nicht weiß.

E10 Wie sieht Ihr Impfkonzept aus? Werden Impfungen vor der Einstallung in die Mast an Ihren Tieren durchgeführt?

- ☐ Mein Impfkonzept steht schon seit vielen Jahren.
- ☐ Mein Impfkonzept bespreche ich mit meinem Tierarzt und passe es gegebenenfalls an.
- ☐ Mein Impfkonzept bespreche ich mit meinem Ferkelaufzüchter und Tierarzt und passe es gegebenenfalls an.
- ☐ Mein Impfkonzept besteht darin, nicht zu impfen, da Impfungen mir zu aufwendig und teuer sind.
- ☐ Andere Antwort: ___________________________
- ☐ Ich möchte dazu keine Einschätzung abgeben.
- ☐ Ich kann dazu keine Einschätzung abgeben, weil ich es nicht weiß.

F Bestandsprobleme

F1 Was ist Ihr hauptsächliches Bestandsproblem und gibt es zudem noch weitere Bestandsprobleme?
(Antwort in Stichpunkten)

F2 Gibt es in Ihrem Betrieb bestimmte Bestandsprobleme, die immer wieder auftauchen? Wenn ja, welche?
(Antwort in Stichpunkten)
