# Supplementary material for: Health scores for farmed animals: Screening pig health with register data from public and private databases
Source: PLoS One. 2020 Feb 4;15(2):e0228497. doi: 10.1371/journal.pone.0228497 (PMC6999879; doi:10.1371/journal.pone.0228497)

Supplement 2: Distribution of the prevalence of meat inspection codes and their z-values in a random sample of 1,747 German pig farms in the second half of 2016

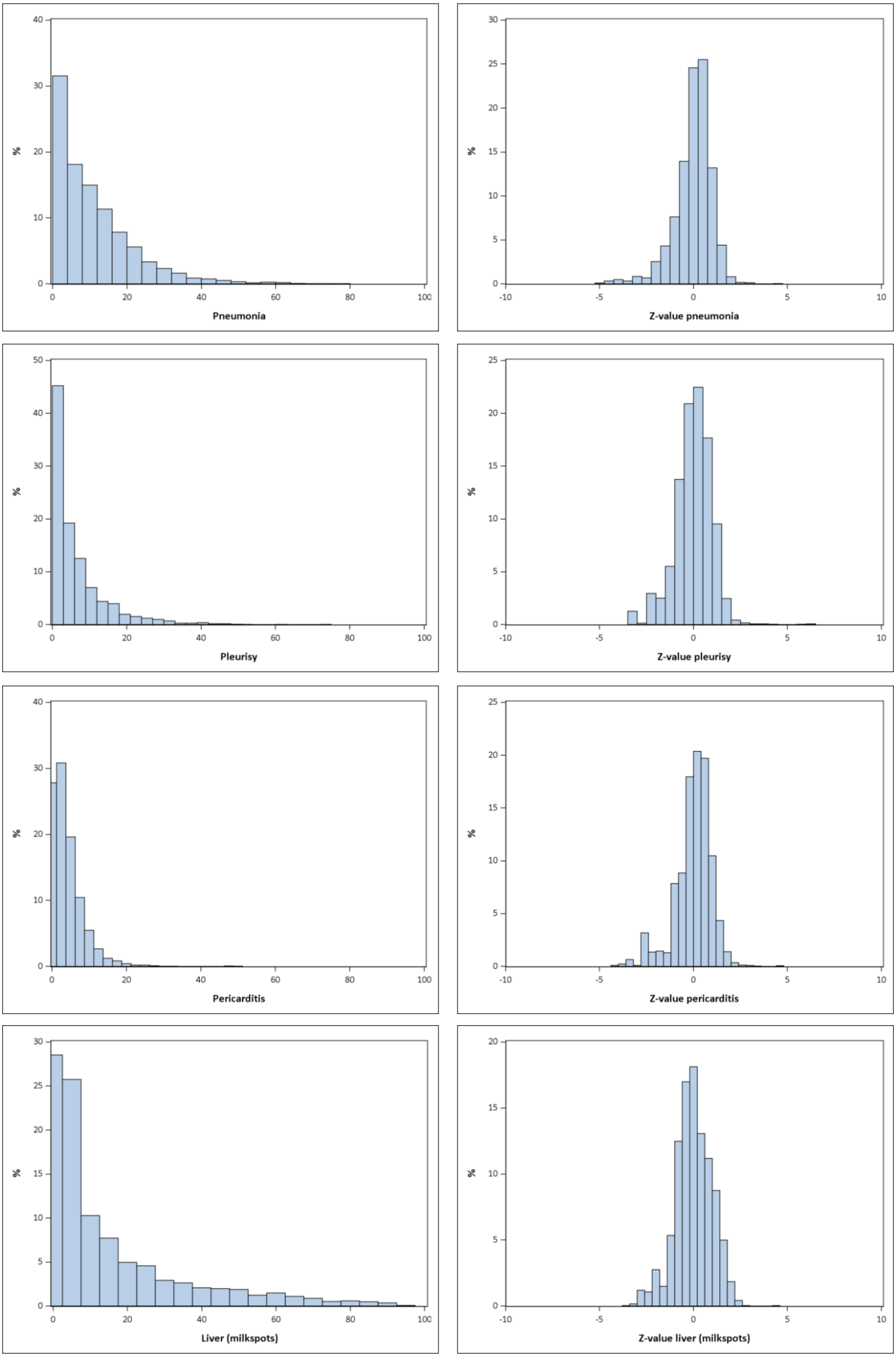

Supplement 2: Distribution of the prevalence of meat inspection codes and their z-values in a random sample of 1,747 German pig farms in the second half of 2016

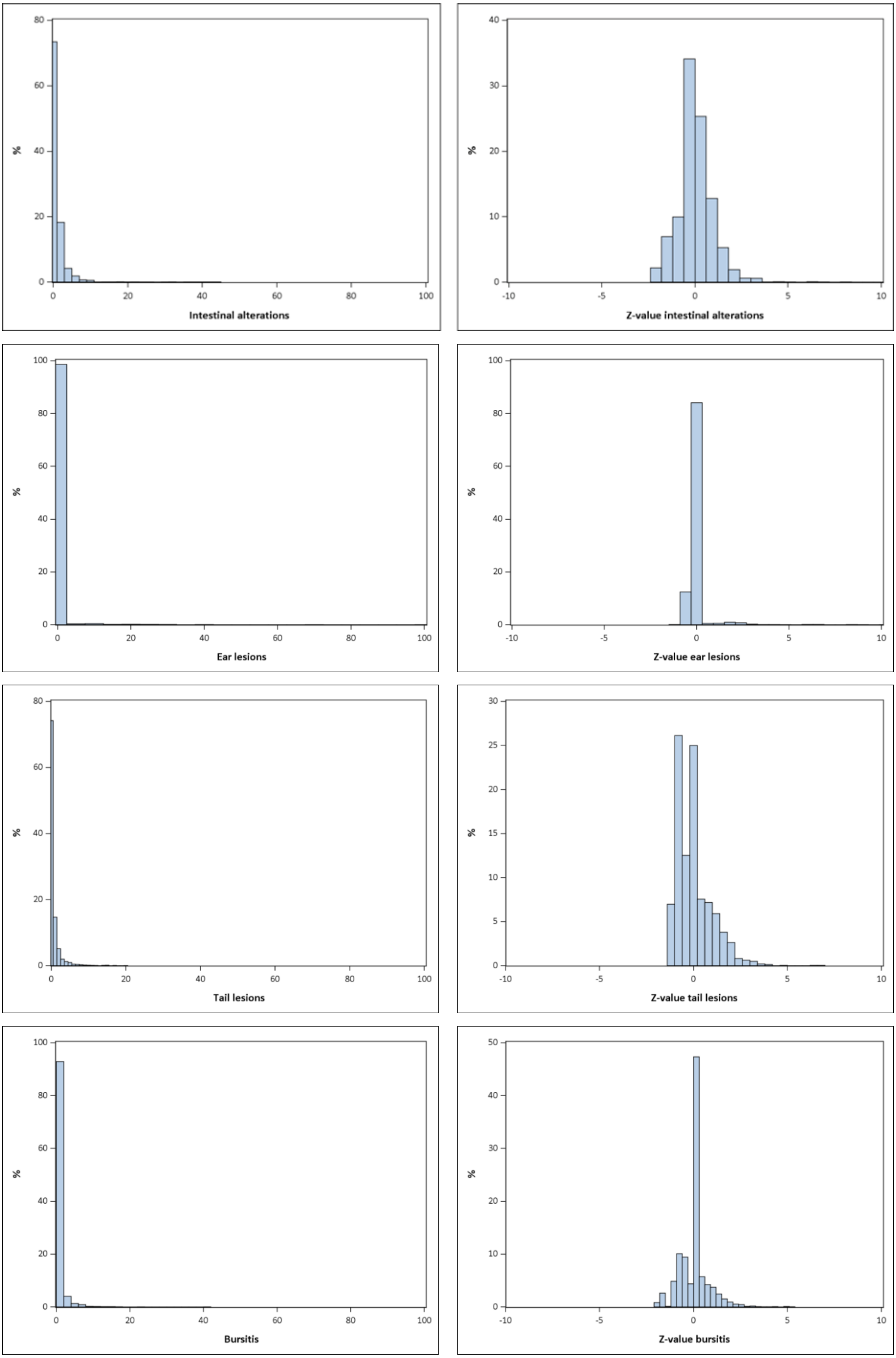

Supplement 2: Distribution of the prevalence of meat inspection codes and their z-values in a random sample of 1,747 German pig farms in the second half of 2016

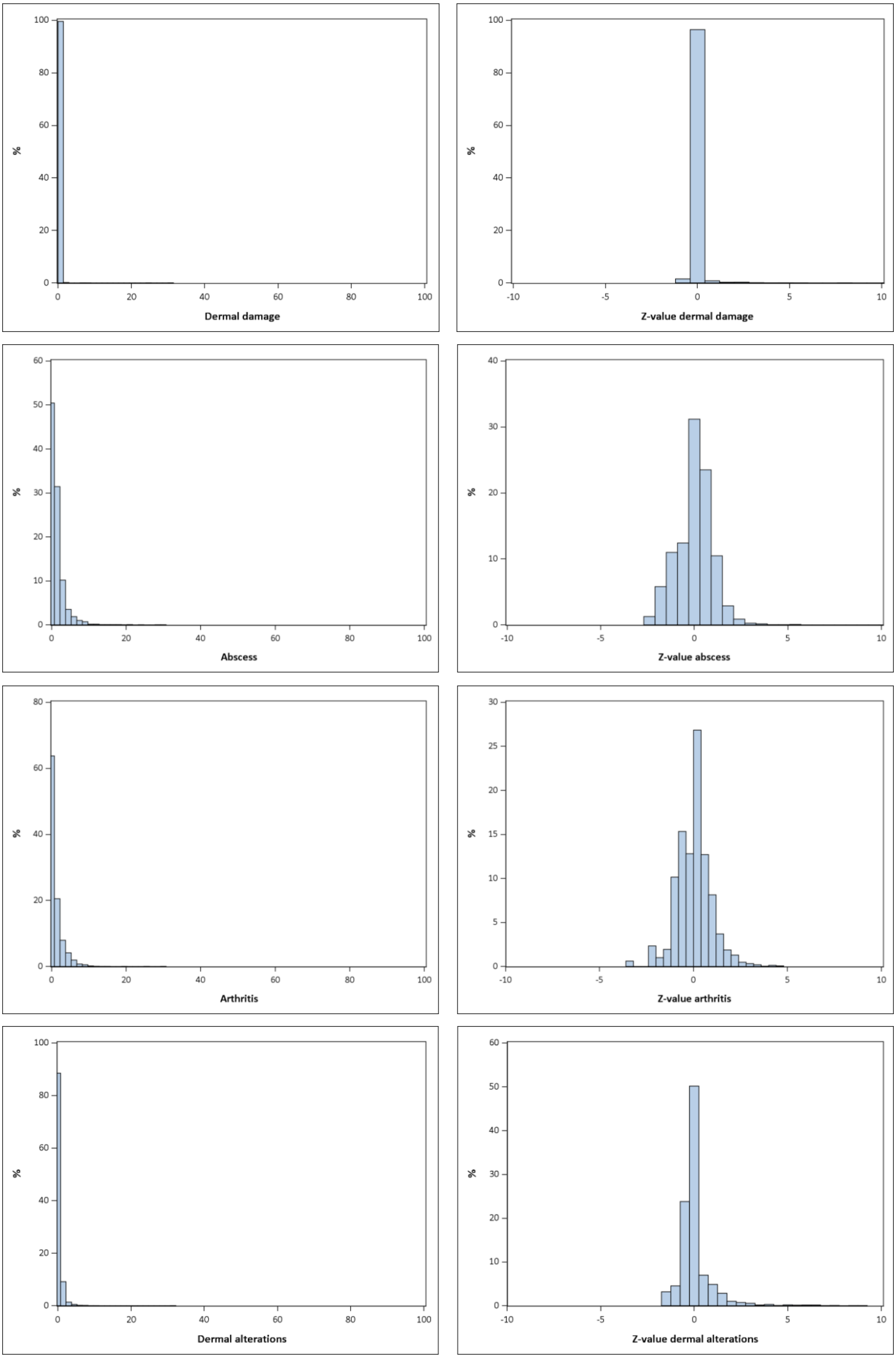

Supplement: S2 Supplement — (PDF) [file pone.0228497.s002.pdf]
